# Policy perspectives on post pandemic influenza vaccination in Ghana and Malawi

**DOI:** 10.1186/s12889-017-4058-5

**Published:** 2017-02-28

**Authors:** Evanson Z. Sambala, Lenore Manderson

**Affiliations:** School of Public Health, Faculty of Health Sciences, University of the Witwatersrand, 27 St Andrew’s Road, Parktown, Johannesburg, 2193 South Africa

**Keywords:** Herd immunity, Pandemic influenza, Vaccine coverage, Vaccination strategy

## Abstract

**Background:**

In the late 1990s, in the context of renewed concerns of an influenza pandemic, countries such as Ghana and Malawi established plans for the deployment of vaccines and vaccination strategies. A new pandemic was declared in mid-June 2009, and by April 2011, Ghana and Malawi vaccinated 10% of the population. We examine the public health policy perspectives on vaccination as a means to prevent the spread of infection under post pandemic conditions.

**Methods:**

In-depth interviews were conducted with 46 policymakers (Ghana, *n* = 24; Malawi, *n* = 22), identified through snowballing sampling. Interviews were supplemented by field notes and the analysis of policy documents.

**Results:**

The use of vaccination to interrupt the pandemic influenza was affected by delays in the procurement, delivery and administration of vaccines, suboptimal vaccination coverage, refusals to be vaccinated, and the politics behind vaccination strategies. More generally, rolling-out of vaccination after the transmission of the influenza virus had abated was influenced by policymakers’ own financial incentives, and government and foreign policy conditionality on vaccination. This led to confusion about targeting and coverage, with many policymakers justifying that the vaccination of 10% of the population would establish herd immunity and so reduce future risk. Ghana succeeded in vaccinating 2.3 million of the select groups (100% coverage), while Malawi, despite recourse to force, succeeded only in vaccinating 1.15 million (74% coverage of select groups). For most policymakers, vaccination coverage was perceived as successful, despite that vaccination delays and coverage would not have prevented infection when influenza was at its peak.

**Conclusions:**

While the vaccination strategy was problematic and implemented too late to reduce the effects of the 2009 epidemic, policy makers supported the overall goal of pandemic influenza vaccination to interrupt infection. In this context, there was strong support for governments engaging in contracts with pharmaceutical companies to ensure the timely supply of vaccines, and developing well-defined guidelines to address vaccination delays, refusals and coverage.

## Background

The 2009 influenza pandemic was the first pandemic to be declared by the World Health Organization (WHO) in the twenty-first century. By 1 August 2010, over 18,449 laboratory-confirmed influenza-related deaths had been reported in more than 214 countries and territories [1]. Yet only 168 deaths were reported in Sub-Saharan African countries [1], with one in Ghana and none in Malawi, reflecting extreme underreporting as countries failed to record or stopped recording individual cases where surveillance and monitoring systems were poor [2]. Simonsen and colleagues [3] and Dawood and colleagues [4] estimate respectively that mortality was 10 to 15 times higher than WHO’s estimate, with some 151,700 to 575,400 deaths in the first year. Of these, around half are estimated to have occurred in southeast Asia and Africa, where the high death rates reflected larger immunocompromised populations due to HIV [5, 6] and inadequate public health infrastructure, poor sanitation, and poor living conditions [6].

Pandemic influenza poses a serious health threat globally [7]. Its occurrence is unpredictable, the virus spreads rapidly in urban areas and through travel, and populations’ lack of immunity to novel influenza strains can result in a rapid spread [8]. The influenza virus constantly mutates, limiting the impact of protection by vaccination, and immunity conferred in one pandemic influenza period will not reliably prevent new infections by an antigenically drifted strain [9]. New vaccines take at least 6 months to develop [10], during which time WHO recommends the use of non-pharmaceutical interventions including increased surveillance and the implementation of such policies as quarantine, border control and hygiene practices [11]. Further, the success of influenza vaccination depends on the timely availability of a vaccine [12], its acceptability and coverage [12], the ages of those vaccinated [13, 14], actual vaccine efficacy and effectiveness [15], and, as we elaborate below, it is negatively affected by operational shortcomings of influenza vaccination programmes including delays in influenza importation and distribution and competing health priorities [16]. A review of national preparedness plans for Ghana and Malawi suggests many tasks of planning for and responding to pandemic influenza through influenza vaccination remained unmet, including those related to influenza surveillance, monitoring and reporting [17, 18].

The global response to the 2009 influenza pandemic involved the production of 900 million doses of pandemic influenza vaccines for distribution in low income countries, with support for their purchase and distribution from WHO and financial aid from high-income countries [19]. Ghana and Malawi were among the countries to receive free consignments of the monovalent vaccine [20, 21], but in both countries, these arrived in May-June 2010, some 8 to 10 months after its first availability in September 2009 and arguably too late in the pandemic to have any significant effect on transmission [21, 22]. As we discuss below, designated high risk groups (health workers, security personnel, pregnant women and children) in Ghana began to receive doses of influenza vaccines by mid June 2010, with H1N1 vaccine deployment continuing into the post pandemic period. The designated high risk groups in Malawi were vaccinated only in April 2011, nearly 10 months after the first shipment of vaccines had been received in June 2010. In this article, we present the results of a study of policymakers in Ghana and Malawi, their views on the introduction of the vaccines, the role of vaccination in prevention and control, and the operational shortcomings that limited their effectiveness as a public health strategy.

## Methods

### Research design

A qualitative research study design, with in-depth interviews, was adopted to evaluate and understand policymaker’s views on vaccination. Ghana and Malawi were identified from among the 46 countries that attended the first African Regional Conference on Pandemic Influenza A (H1N1) in 2009, in which the first author took part. The two countries were purposively selected based on economic status, geographical location, influenza surveillance systems, and the availability of a national pandemic preparedness plan. These considerations included a number of points of comparison. Ghana is a lower-middle income economy; Malawi a low income country with especially limited health expenditure. Although both countries have health systems based around primary health care, Ghana’s system is better established, with a national influenza centre and national influenza laboratory, and with better hospitals and trained professionals than Malawi. Ghana and Malawi were among the first countries in Africa to have developed pandemic plans. In addition, Ghana and Malawi were feasible and practical to collect data since both are English-speaking countries, politically stable and safe, allowing for interviews and data analysis to be conducted without translation assistance. The comparison between and triangulation of information derived from these two settings provide the basis for substantiation and validity [23].

### Participants and sampling

Policymakers eligible for the study included people in country Planning for and Response to Pandemic Influenza (PRPI). At the African Regional Conference on Pandemic Influenza A (H1N1), held in August 2009 in Johannesburg, South Africa, four potential interviewees from Ghana and three from Malawi attending the conference were invited to recommend colleagues, resulting in an overwhelming response: sometimes one person would recommend between three and five people. To ensure a representative sample that would reflect policymakers’ views, we wrote to the Ministry of Health in Malawi and the Ghana Health Service and Ministry of Health in Ghana requesting a complete list of experts involved in PRPI, including those involved in drafting the pandemic plans. The number of participants identified through this process was inadequate to reach a representative sample, since most of those identified were high level national experts rather than local, regional or lay policymakers working at district levels. Consequently, the first author contacted district and regional health offices to identify further participants. These included politicians, environmental health officers, medical and nurse directors, veterinary officers, executive directors, scientists, researchers and managers, working within government, civil society, non-governmental organizations, and multilateral agencies including WHO (Table 1).

**Table 1:** Institutions included and excluded in the study

Ghana interviews = 24	Malawi interviews = 22	
1. Ghana Health Service (GHS) Surveillance Department 2. GHS, Public Health 3. Ministry of Food and Agriculture (MOFA), Veterinary Services 4. MOFA, Veterinary Services 5. MOFA, Veterinary Services 6. GHS, National Surveillance Unit 7. Noguchi Memorial Institute for Medical Research (NMIMR) 8. GHS, Upper West Health Directorate 9. National Disaster Management Organization (NADMO) 10. GHS, Management Information Systems 11. World Health Organization (WHO), Expanded Programme on Immunization (EPI) 12. WHO, EPI 13. GHS, Greater Accra Health Directorate 14. GHS, Ashanti Health Directorate 15. Ghana Red Cross Society (GRCS) 16. World Bank, Ghana 17. USAID, Ghana 18. UNICEF, Ghana 19. Ministry of Lands, Forestry and Mines (MLFM) 20. Food and Agriculture Organization (FAO), Ghana 21. Department of Defense (DOD) 22. Quality Health Partners, Ghana 23. GHS, Child Health Team 24. Politician, Ghana	25. Department of Disaster Management Authority (DODMA) 26. Care International, Malawi 27. Ministry of Health (MoH), EPI National Task Force 28. FAO, Malawi 29. Malawi Red Cross Society (MRCS) 30. Nations Newspaper 31. College of Medicine, UNIMA 32. MoH, Preventive Health Services 33. MoH, Community Health Science Unit 34. MoH, Pandemic Preparedness Unit 35. Ministry of Agriculture (MoA), Avian Influenza 36. Blantyre City Council, Health 37. World Bank, Avian Preparedness 38. MoA, Animal Health 39. Malawi Police Service 40. Catholic Development Commission in Malawi (CADECOM) 41. Lilongwe City Council, Health 42. USAID, Avian Preparedness 43. MoH, Kamuzu Central Hospital 44. United Nations High Commissioner for Refugees (UNCHR), Malawi 45. Lay Person, Rumphi District 46. Malawi Army, Health	
Institutions excluded from the study = 15	Declined interview Ghana & Malawi = 5	Institutions not interviewed due to data saturation = 4
47. Ghana Education Service (GES) 48. Ministries of Water Resources Works and Housing MWRWH, Ghana 49. Politician and advocate, Ghana 50. GHS, Public health 51. GHS, Child Health Team 52. GHS, EPI and Logistics 53. Ghana Police Services 54. Department of Wildlife, Ghana 55. Episcopal Conference of Malawi (ECM) 56. World Vision, Malawi 57. Malawi Council of Churches 58. Ministry of the Interior, Ghana 59. Lay Person, Ghana 60. Lay Person, Malawi 61. National Health Sciences Research Committee (NHSRC), Malawi	62. Politician, Ghana 63. GHS, Communicable Diseases 64. Centre for Disease Control and Prevention (CDC), Ghana 65. GHS, Volta Health Directorate 66. MoH, Malawi	67. Korle-Bu Teaching Hospital, Ghana 68. GHS, Epidemiology and AIDS Department 69. GHS, Planning Department 70. Catholic Health Association of Malawi (CHAM)

Numbers in the table represent participants identified through snowballing process

Seventy potential participants were identified in Ghana and Malawi, but 15 were subsequently excluded because they did not meet the selection criteria. All eligible participants were contacted with an official letter, information sheet, and consent form, sent either by email or fax. Both verbal and written consent was required in order to take part in the study. Five policymakers did not respond and eventually, 46 participants were interviewed (*n* = 24 Ghana, *n* = 22 Malawi, see Table 1). Data generated from this were sufficient to achieve saturation, when no new themes or new information were generated.

### Data collection

In-depth interviews were used to collect primary data, and were complemented by a review of the pandemic plans of the two countries, using the six thematic areas set out in the WHO checklist [24]: planning and coordination, surveillance, communication, public health interventions, patient management, and maintaining essential services. This enabled us to check and compare respondents’ views on the levels of preparedness, strengths and gaps for each country. Data from the pandemic plans were used to formulate interview questions. The interview guide used in Ghana and Malawi was similar, allowing us to identify variations or overlaps if any existed between the two countries, and included the following key questions: (1) How did the countries prepare and respond to the H1N1 pandemic? (2) Were there delays in vaccination programmes, and for what reasons? (3) What were the challenges encountered during the vaccination period? (4) What was the role of the vaccines and vaccination in preventing the spread of infection? (5) What was the understanding of post pandemic influenza vaccination coverage using a pandemic vaccine? and (6) What were the perceived benefits of vaccinating in the post pandemic period?

In both countries, interviews were mainly conducted in the interviewees’ offices or in cafés and hotel lobbies, lasting on average 50 min. Interviews were conducted in January – May 2012 in Ghana, and September 2012 – January 2013 in Malawi, with time between the two countries for reflection and provisional analysis of the data. All interviews were tape-recorded and transcribed verbatim. Ethics approval was obtained from the Medical School Research Ethics Committee of the University of Nottingham, UK and the Ministry of Health in Ghana and Malawi to ensure the highest integrity and quality of the study.

### Data analysis

Data analysis was ongoing from the first interview, with the first author listening and re-listening to the audio recordings to gain familiarity with the data and allow for iteration, with notes entered into a data analysis logbook. All transcribed interviews were exported to NVivo 8 to facilitate the coding and retrieval process, and organised as cases. For example, respondents from Ghana and Malawi were labelled as case nodes with reference to the organization represented. During coding, text was examined closely, line by line, prior to generating codes and analytic themes. Preliminary themes that did not fit particular data extracts were redefined or discarded. The final phase involved searching selective coding, with re-emerging themes further defined and refined. Data extracted were eventually coded for preparedness, responses, herd immunity, perceived benefits of vaccines, vaccine coverage, timing of vaccination, vaccination strategies, refusal of vaccines, and politics, as we elaborate below.

## Results

In total, as noted above, 46 out of 50 participants were interviewed in Ghana (24) and Malawi (22), representing a 90% response rate to original invitations to participate. Building on the codes generated during analysis, seven main themes were identified: (1) pandemic preparedness and responses (2) vaccine coverage (3) late timing of vaccination and the politics that influenced this, (4) perceived herd immunity (5) perceived benefits of vaccine in relation to seasonal influenza, (6) refusal to vaccinate, and (7) frustrations with vaccination strategies.

### Pandemic preparedness and response

**Fig. 1:**
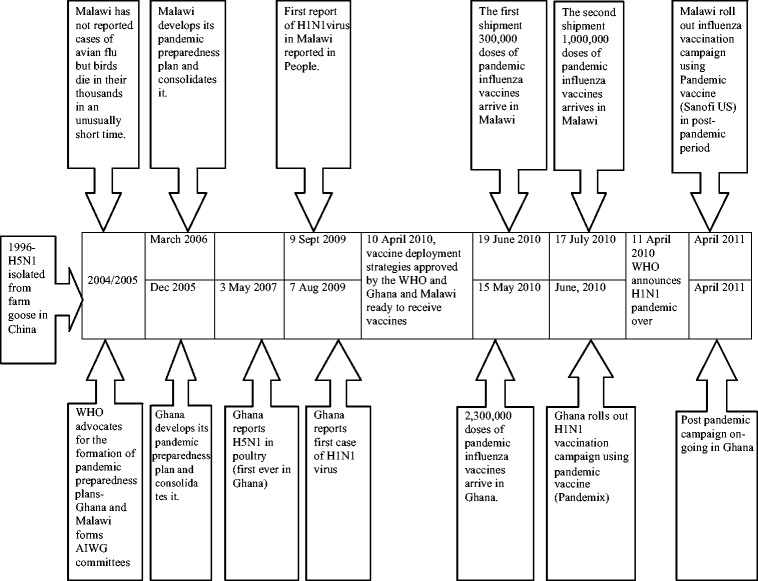
Timeline of pandemic influenzas and significant milestones in Malawi and Ghana

Figure 1 provides a timeline of H5N1 (avian influenza) and H1N1 (pandemic influenza) outbreaks in Ghana and Malawi, and the significant milestones achieved in terms of preparedness and responses.

Preparation prior to the 2009 pandemic influenza in Ghana and Malawi evolved around the six sub-themes of preparedness, i.e. prevention and containment, health system, coordination, influenza surveillance, communication, and maintaining essential services. As reflected in the national plans, national pandemic preparedness policies in Ghana and Malawi were deployed to respond to the 2009 pandemic influenza. Both countries planned to prevent and contain influenza by using non-pharmaceutical interventions (quarantine, closing schools and other public places, isolation of suspected cases and border control) and pharmaceutical interventions (antiviral drugs, pandemic vaccines and antibiotics). Both countries planned to mount early surveillance systems to detect the threat of pandemic and to determine human resource and operational capacity to respond to this. This involved establishing communication and reporting schedules. Other planning efforts involved strengthening leadership capacity and identifying funds for the implementation of the pandemic plans, including for influenza education, human resources and laboratory surveillance. Emphasis was also placed on frequent interactions among lead agencies, partners and local and international stakeholders. Non-health preparations, such as a business contingency plan and ethics plans, were not part of the national preparedness.

Both countries prepared for the deployment of pandemic influenza vaccines and vaccination, However, in both cases, vaccination strategies were weak, for example, failing to account for specific issues around vaccine procurement, storage, distribution, liability, and licensing. While both countries specified the need to acquire antiviral drugs such as oseltamivir and zanamivir, and masks and gowns, neither anticipated challenges relating to the scarcity of vaccines and other pharmaceutical products.

While seasonal influenza is prevalent and a major economic burden, neither Ghana nor Malawi included within the vaccination strategy a plan to vaccinate seasonally. In addition, they did not include detailed plans for the recruitment of temporary or additional health workers and volunteers were pandemic influenza vaccination required, or to care for people infected during a pandemic. Plans did not address how health care delivery systems might manage care in limited resource settings – for example, how to maintain existing centres for influenza immunization or when to close vaccination clinics, when real-time information on supplies of and demand for influenza vaccine doses were unavailable. Neither country included plans for police to maintain peace and order, nor to provide quarantine facilities for travellers. Communication with local stakeholders such as chiefs, private hospitals and mortuaries, was not addressed, and arrangements for home care for patients were not considered. Further, neither country considered public-private partnerships that might be necessary for continuity of essential services such as water, energy and safe transport.

To validate country responses, respondents were asked how they had prepared and responded to the 2009 pandemic influenza. Respondents drew on diverse discourse to explain the varying roles: some focused on measures undertaken to reduce the effects of pandemic influenza, such as antiviral drug treatment during and post pandemic; others recounted that implementation failed to account for the delivery of effective risk communications to prepare the public to respond. One of the most frequent themes in the interviews, in both Ghana and Malawi, was that implementation of pandemic preparedness and responses was “clumsy” and “incomplete.” One policymaker representing a regional office of the Ministry of Health in Malawi said, “I say always that our responses to pandemic influenza were frustrating and lacked clarity.” A policymaker from Ghana Health Service remarked that “we (the health service) did not do well, but our responses to the pandemic influenza were as satisfactory as the assumptions on which they were proposed.”

Despite the numerous challenges and the frustration that policymakers expressed, many milestones were achieved in response to the pandemic. For example, both Ghana and Malawi quickly set up special advisory committees on pandemic influenza within their health ministries to oversee the implementation of the pandemic plans. As part of the responses effort, Ghana and Malawi participated in the African Regional Conference on Pandemic Influenza A (H1N1) in Johannesburg, as noted above. In addition, both countries entered into a formal agreement with WHO for the deployment of donated H1N1 vaccines on 10 April 2010, after submitting a letter of intent (LOI) to the Director General of WHO in response to a call for this on 22 September 2009. After receiving the vaccines, Ghana acted quickly to vaccinate select groups in a relatively short period of 4 weeks. Malawi, however, was able only to administer the vaccinations over 200 days after the vaccines had been delivered. Earlier in the epidemic, however, the Ministry of Health in Malawi developed a partnership with a reference laboratory in Kenya to send early signals to ensure a health service response. Ghana participated in the Influenza Surveillance Network (FluNet), a global tool for surveillance setup by the WHO, to report and share its limited influenza real-data electronically with the rest of the world.

Despite the lack of laboratories and capacity for surveillance, through the Integrated Disease Surveillance and Response (IDSR) system, Malawi established surveillance at ports of entry, with routine checks for suspected cases and suspected samples sent to Kenya for diagnostic confirmation. These were returned after 2–3 weeks. Through this process, Malawi was able to register four confirmed cases and no deaths. Surveillance response in Ghana was better because Ghana had surveillance infrastructures such as the Noguchi Memorial Institute for Medical Research (NMIMR), which provides oversight of the national influenza centre in Accra, Ghana. Two staff from NMIMR were trained in Cairo, Egypt in influenza diagnosis and virus characterization. During the pandemic influenza period, NMIMR identified six sites for sentinel surveillance of influenza viruses and procured a real-time PCR machine and reagents to improve data reporting. Surveillance of pandemic influenza and monitoring were conducted at borders of entry during the post and pandemic periods. Schools were closed. For example, Lincoln Community School, located in Accra, Ghana and the site of a localised outbreak with two percent of 700 students identified as confirmed cases, was closed to break the cycle of transmission. A total of 345 specimens (294 suspected cases and 51 contacts) were investigated at the NMIMR, with 38 confirmed as positive for the H1N1 virus.

Communication about the disease was promoted through various communication channels such as radio, TV and leaflets. In Ghana, telephone hotlines were established to inform the general public on issues relating to influenza, such as protocols and triage decisions regarding access to limited vaccines in moderate and worst-case scenarios. In Malawi, communications messages initially mainly targeted poultry farmers against the H5N1 subtypes, but during the pre-pandemic period, as part of a new strategy, switched to the 2009 H1N1 pandemic and targeted health workers and the general population. Policymakers who were interviewed emphasized that responses to pandemic influenza should occur in a *timely manner* on *what is known* about the disease, in order to generally improve communication, public knowledge and perception about the disease.

Neither country communicated with the public on how soon vaccines would be made available or how effective they would be against the virus. A policymaker representing the Ghana Health Service commented that “the likely nature or duration of the pandemic influenza, its spread, its peak and decline” was never sufficiently stressed and “nor did officials sufficiently inform the public on the use and safety of vaccines.” Respondents expressed a deep sense of dissatisfaction over the pandemic influenza response to the outbreak and the inability of their governments to respond effectively. All respondents talked about how responses were affected by lack of critical infrastructure for preparedness such as surveillance and financial resources, and the lack of operational resources. As a result they characterised national strategic plans on pandemic influenza as “inflexible” and “unresponsive” to many operational aspects, including maintenance of essential services, patient management services, and public health action.

### Vaccine coverage

Theme two focused on the donation of vaccines to Ghana and Malawi from the WHO, sufficient for 10% of the risk population of the respective countries. Policymakers were asked about the number of people vaccinated with the 2009 pandemic vaccine. In Malawi, around 1.15 million people, mostly pregnant women and children, were vaccinated, and around 75% of the supplies from WHO were used for this purpose. The remaining 25% of the WHO supplies were unaccounted for. Policymakers in Ghana maintained that they had used all the vaccines supplies donated by the WHO, ensuring a 100% vaccine coverage of the risk population with 2.3 million doses of pandemic influenza A (H1N1) pdm09 vaccines targeting health workers, security personnel, pregnant women and children. The decision to target “at risk” groups was based on the WHO Strategic Advisory Group of Experts (SAGE) on immunisation which recommended vaccinating health care workers as first priority to protect the essential health infrastructure, and then vaccinating to reduce morbidity and mortality among pregnant women, children over 6 months to 11 years of age, and vulnerable people including those with chronic diseases (Table 2). Given the coverage needed for vaccines to prevent the spread of infection, this low coverage would not have been effective, as it would not have served the affected population.

**Table 2:** Target groups and deployed vaccination strategies against pandemic influenza

Vaccination Strategies in Ghana and Malawi		
1. To vaccinate within 7 days after receiving the vaccines. 2. To vaccination in phase i.e. to cover 2% of the population followed by 8%. 3. To first vaccinate 2% of HCW to protect the integrity of the health-care system and the country’s critical infrastructure. 4. To reduce morbidity and mortality among pregnant women. 5. To reduce transmission of the pandemic virus within communities by vaccinating children. 6. To undertake public awareness campaigns through TV, radio and print media.		
Target groups	Ghana (100%)	Malawi (75%)
Health-care workers (HCW)	Vaccination teams visited each health facility after an education campaign. More campaigns undertaken to improve uptake. HCW and security personnel voluntary got vaccinated first without incentives.	Vaccination teams visited health facilities accompanied by an educational campaign. Due to low uptake, vaccination teams deploy the police force
Pregnant women	Vaccination teams visited maternal clinics and held one to one sessions. Programme supported Information, Education and Communication (IEC) to change attitudes and perceptions. More campaigns undertaken to improve uptake.	Vaccination teams visited maternal clinics accompanied by an educational campaign. More Information, Education and Communication (IEC) campaigns to change attitudes and perceptions. Due to low uptake, vaccination teams deploy the police force.
Children aged 6 months to 11 years:	Mobile vaccination teams visiting nurseries and schools supported by Information, Education and Communication (IEC) on attitudes and perceptions. More campaigns undertaken to improve uptake.	Vaccination teams visited under-five clinics accompanied by an educational campaign. More Information, Education and Communication (IEC) campaigns to change attitudes and perceptions. Due to low uptake, vaccination teams deploy the police force.
Population Immunocompromised (HIV/AIDS) and vulnerable people such as the elderly.	Vaccinated via routine antenatal clinics at all levels of health care delivery. More campaigns undertaken to improve uptake.	Not targeted

Most policymakers stated that the quantity of vaccines that they received fell well short of the supply needed to enable acceptable coverage to stop the virus from circulating. For most policymakers, procurement of enough vaccine supplies was hampered by lack of clear planning on vaccination. A senior officer from the Food and Agriculture Organization in Ghana acknowledged: “Honestly, do we even have a vaccine strategy? Because the one I have seen is poorly designed, unable to support efforts to secure enough pandemic vaccines or improve vaccine uptake.”

Many policymakers believed that their countries lacked a vital information system and operations capabilities that would support the deployment of vaccines to a large group of people, even if limited to target groups such as pregnant women and children. In addition, there were no debates within government on the best way to coordinate vaccination programmes to ensure on-time availability and high coverage. Policymakers from the Ministries of Health in both countries referred to weak planning systems in the national preparedness plans and the lack of interest by the government to invest in vaccine research, development and production, which might have facilitated the management and execution of the vaccine deployment operations.

### Politics and timing

Most policymakers in Malawi identified operational gaps specifically around vaccinating the target population late in the post pandemic period. All policymakers agreed that vaccination started late, and while the purpose of the vaccine was to protect people from acquiring the disease during the pandemic period, this did not happen since the disease had abated at the time of vaccination. Reviews of the weekly and monthly distribution of cases suggest that the majority of cases of H1N1 (90%) was reported in 2009, reaching its peak during August and September; only 10% of lab confirmed cases occurred in 2010 [22]. The vaccines were delivered late mainly due to the late release of operational funds from WHO, competing priorities, and the reduced severity of the pandemic. Neither Ghana nor Malawi had direct access to vaccines, and so relied on donor countries to make vaccines available to the WHO for countries that could otherwise not afford the vaccine. The vaccine pledges to the WHO also depended on donor countries satisfying their local requirements to meet their own vaccination targets before they were able and willing to assist poor countries; in addition, availability sometimes depended on vaccine production schedules. A policymaker working as a district health officer in rural Malawi also alluded to the late arrival of vaccines as a result of the WHO preconditions for supply of vaccines: “You know, before the WHO donates vaccines, they demand that recipient countries apply and meet the preconditions for supply of vaccines within the WHO Deployment Initiative.” A policymaker representing a non-health department concerned with disasters in Malawi wondered how such a country without a strong legal framework to support vaccine licensing or registration could be required to meet these preconditions for supply of vaccines in a pandemic situation.

The reasons for the late arrival of vaccines and implementing pandemic influenza vaccination programmes were said by respondents to have been driven by “politics” and “hidden agendas.” Three policymakers from Ghana and Malawi believed that pharmaceutical companies were paid in advance by rich countries to produce vaccines for poor countries; thus the pharmaceutical companies had an obligation to produce and supply the requested vaccines to poor countries regardless of when they could do so. These policymakers believed that their governments had then to deliver the vaccinations for “financial” and “political” reasons. For example, a policymaker from Malawi cited that through certain public health officials and politicians, his government had pursued post pandemic influenza vaccination in order to obtain money from the WHO, which was largely spent on training, transportation and per diems associated with the vaccination programme. In other instances, vaccination programmes were seen to be influenced by foreign expertise, external funding and foreign policies. In general, most representatives working in government and NGOs in both countries blamed their governments for the late acquisition of vaccines and the failure of the WHO to speed up delivery from September 2009 when the vaccine was available. A senior representative policymaker in the Ministry of Health in Malawi captured this vividly: “It was unexpected that the Malawi government and WHO could not arrange or deliver vaccines until July 2010 and kept them until April 2011, arguably well into the post pandemic period.” Representatives from the WHO from both countries considered the failure to make vaccines available on time was due to the complex legal systems related to liability, regulatory registration and vaccine licensing. For instance, the role of licenses for vaccine use changed outside the pandemic period, and the WHO demanded that the recipient countries register the vaccine with national regulatory bodies before the product could be imported or distributed. In normal circumstances in Ghana and Malawi, drug registration could take a long time and significant resources. Most policymakers were aware that vaccines might not arrive on time. A policymaker from a humanitarian organization in Malawi emphasized this: “I know during the first few months there will be no vaccines, and this is the reason vaccination is not the primary intervention method in dealing with pandemic influenza.” Policymakers expected that their national planning systems would have attempted to secure vaccine supplies in time for their population by engaging in possible contracts with pharmaceutical companies. A representative from WHO, Ghana, explained that “if the government can’t clearly demonstrate that it is trying to help its people, then how do you expect foreign partners to help you?”

### Perceived herd immunity and benefits for seasonal influenza

When specifically asked about the vaccination coverage of the 10% “risk group” against the H1N1 pandemic influenza, many policymakers commented that “vaccines save lives,” especially if used rapidly and widely once the vaccine is available. For example, one policymaker from the Ghana Health Service stated, “I am sure vaccines are among the proven strategies to address an influenza pandemic if they are made available on time and widely.” Most policymakers suggested, but did not directly express, that the 10% of risk population vaccine coverage in a given country would be adequate to achieve herd immunity. On the other hand, a representative from World Bank, Malawi, who was confident of the role of a vaccine to prevent infections, explained: “It’s not rocket science, but vaccines are vital to stop the spread of influenza during the pandemic.” But the benefits of the vaccines were minimal and questionable because they were offered well after the disease had abated.

Some policymakers, who thought that the vaccines were beneficial despite timing of their delivery, related their usefulness to achieving protective benefits. As a representative from United Nations High Commissioner for Refugees (UNHCR) in Malawi explained, “of course, it was difficult to convince everybody on the vaccines’ benefits related to indirect risk reduction associated with herd immunity.” A senior policymaker from the Ministry of Food and Agriculture, Ghana, involved with H5N1 pandemic planning, commented that “herd immunity was possible but would require a lot of vaccines to go around – more than 10% of the risk population – and depended on whether the government could afford it.” A few policymakers commented that while wide vaccine coverage was important, it would not have attained herd immunity and so stop the virus from circulating; this would have required everyone’s cooperation in being vaccinated.

Apart from the benefits of post pandemic vaccination against H1N1 influenza in reducing symptomatic cases and deaths, participants drew attention to the perceived benefits of vaccines, after the pandemic period, against seasonal influenza virus. One policymaker with the Community Health Sciences Unit, Malawi, commented: “As far as I know it provided protective effects in susceptible persons against the viral strain of influenza matched to the vaccine that was circulating as seasonal influenza.” The 2009 H1N1 virus was antigenically unchanged in the 2010/11 season, and it is likely that it would have infected vulnerable groups in the form of seasonal influenza. There was general consensus that the vaccines rendered benefits among those vaccinated, but this depended on other factors such as vaccine efficacy, effectiveness, age of person vaccinated, and their immunological status.

### Refusals and enforcement

The H1N1 vaccine deployment activities in Ghana and Malawi continued for a period of over 6 months from receiving the vaccine. The distribution of vaccine was planned to follow the routine Expanded Programme on Immunizations (EPI) distribution system supported by the EPI national task force in coordinating the country response to the pandemic.

Vaccination refusal emerged as a theme among respondents, in relation to the willingness of people identified as “risks groups” to be vaccinated. Nearly all policymakers interviewed commented on people’s resistance to vaccination, and two thirds of policymakers recounted their struggles to convince people identified as at risk of the importance of being vaccinated. One policymaker working for Noguchi Memorial Institute for Medical Research, Ghana, recalled: “I think people had negative attitudes towards the vaccination programme because they lacked information about the influenza vaccine and the perceived health risk associated to these [vaccines].” Another senior policymaker with the Ghana Health Service maintained that recipients’ reasons for refusal were that they didn’t understand why they had to be vaccinated after the disease had ended.

Most policymakers believed that refusal to be vaccinated related to multiple uncertainties regarding the safety of the vaccines. For example, recipients who were healthy feared that if they were vaccinated, they would “fall sick,” and there were rumours that the vaccine could lead to major complications such as “infertility” or “insanity.” As a district health officer in Ghana narrated, “aahh … people from rural areas think these vaccines are birth control medicines whose only purpose is to stop families from child bearing.” Most policymakers in Malawi cited that people refused vaccination on “religious grounds” and because of “cultural beliefs.” Most importantly, there was mistrust in the vaccination programme and particularly the authorities. Misinformation and unfounded fears in Malawi followed from the Mass Drug Administration (MDA) programme to treat and prevent the further transmission of schistosomiasis and helminth infections, including the alleged death of a school boy after taking the drug praziquantel. After this incident, there was widespread concern and cries of outrage at the severe adverse effects of the drug experienced by school children in a number of schools across the country.

Despite vaccine refusals, both countries achieved very high rates of vaccination. At the start of the programme, low uptake of vaccines was common especially among religious groups such as members of the Zion Apostolic Church. To increase uptake, the Malawi government called in the police to assist with the vaccination by gunpoint, which one policymaker from the Ministry of Health explained as necessary “to be done.” The strategy in Malawi involved forcing targeted recipients to get vaccinated against their will or, as most policymakers described it, “threatening them with the police.” A few policymakers from Malawi believed this would help overcome refusals. However, most policymakers believed that the forced implementation of vaccination risked stigmatising the populations they were meant to protect and that the intimidation of targeted populations to enforce vaccination would have long term problems. One policymaker from the College of Medicine, Malawi explained: “I don’t get decisions that force unwilling recipients using threats to participate in what should be voluntary vaccination to increase uptake. (This) was not a good idea and would damage the good reputation of the country health service.”

In Ghana, despite initial low uptake, there was no violence, and vaccines were not administered to reluctant members of the population by force. Most policymakers in Ghana recounted struggles to reach the target groups, but this was resolved by educating target groups on the need for vaccines using media, pamphlets and one-to-one sessions. These techniques led to improved uptake. One policymaker from the Ghana Health Service reiterated that it was important to encourage target groups to come forward for vaccination without incentives or force.

## Discussion

In this article, we have presented policymakers’ views on vaccination in the context of preventing transmission during the post pandemic influenza period, and evaluated the concept of vaccinating target groups after the influenza infections had abated. The pandemic vaccines only became available to recipients in Ghana in June 2010, and the deployment of vaccines continued over 6 months longer than the time desired in pandemic situations. The pandemic vaccines were available in Malawi in July 2010, but were only used from April 2011, nearly 2 years after the beginning of the 2009 pandemic influenza outbreak. The general policy of WHO is to deploy vaccines and supplies within seven days of the arrival of vaccines [22]. Delayed vaccination inevitably reduces the effectiveness of pandemic influenza control.

For example, policymakers cited the initial refusal of “at risk” groups to be vaccinated due to the late availability and administration of the influenza vaccination. The use of the pandemic influenza vaccines to prevent pandemic influenza would have been effective only if they had been administered early in the pandemic. By April 2011, the pandemic had abated and the impact of the vaccine on reducing serious morbidity and mortality would have been minimal, although the delayed vaccination might have protected against the pandemic influenza virus if it were actively circulating as seasonal influenza in the post pandemic period.

Recent studies highlight the importance of timely vaccination against influenza among at-risk populations to maximize the public health benefits [14, 25]. Borse and colleagues [12] have shown in the US that the effectiveness of the influenza vaccine in reducing symptomatic cases at population level was greatly influenced by the timing of vaccine availability in relation to the timing of disease activity. In Ghana and Malawi, however, as we have described, vaccinations did not start in a timely manner. This is unlikely to change in the near future. On the basis of the pandemic plans for both countries, the vaccination strategy remained unmet and fell short of implementation drills or simulations to test plans for an imminent threat of a future pandemic influenza. The lack of resources to purchase vaccines suggests that these countries will continue to rely on donations; these often come with preconditions and complex legal requirements around liability, regulatory registration and vaccine licensing. We do not suggest waiving or lowering these much-needed preconditions for the supply of vaccines to ensure quality and safety, but governments might need to streamline and expedite the negotiation process, firstly by aligning their country legal frameworks with the global legal systems.

Policymakers believed that delays in vaccination could be reduced significantly if their governments engaged in contracts with pharmaceutical companies to supply vaccines, rather than relying on WHO donations when needed. This is consistent with a well-funded and structured strategy to ensure the rapid procurement of vaccine supplies and delivery to the points of vaccination [26]. Because vaccination efforts were severely hampered by resources at the time of the 2009 pandemic, policymakers suggested shifting planning priorities from being fully committed to addressing the consequences and effects of the pandemic, to preventing it from occurring in the first place, through border management and cluster control. This would require routine surveillance activities such as sentinel reporting of influenza-like illness (ILI) and severe acute respiratory infections (SARI) using various approaches such as outpatient illness surveillance, a pyramid of severity, and the use of RT-PCR machines or a rapid influenza diagnostic test (RIDT) to identify early warning signs of new events [27]. This would improve the operational capacity to assess, monitor and track surveillance data relevant to determine the mortality, attack rates and admission rates of influenza, especially in countries that reported low cases. Epidemiological data for seasonal influenza could be used as a predictive indicator to aid estimates of vaccines needed for pandemic influenza vaccination and additional capacities required to address the pandemic.

The implementation of pandemic influenza vaccinations were slow and frustrating in Ghana and Malawi, with the strategy perceived to have failed because vaccine recipients were not adequately informed about the vaccine efficacy and effectivess, its benefits and possible safety risks. Within pandemic influenza vaccination programmes, recipients need to be made aware about the effectiveness and safety of vaccines, with clear advice that vaccines cannot be 100% safe and effective. Exposing simple and open information on the benefits and risks to recipients may assist with compliance and people’s willingness to be vaccinated. In childhood immunisation programmes, recipients are fully informed, thus there is high acceptance and coverage [28]. Pandemic influenza vaccination is not a routine intervention; it is a relatively new approach of targeting a diverse group of people including children. The lack of information on the safety of pandemic vaccines, and the logic for delayed vaccination, justifies why targeted risk groups were not interested in being vaccinated once the pandemic had ended. Pregnant women and health care workers were initially hesitant because they felt that they were being used as guinea pigs for pharmaceutical interventions.

In this study, most policymakers were aware of the mistrust, doubts and concerns among people in relation to delayed influenza vaccination. While this affected vaccine acceptability, because of the contracts signed with the vaccine donor, the governments of Ghana and Malawi both felt they were obligated to vaccinate at any cost. Accordingly, pandemic influenza vaccination took place either by convincing or forcing the target risk groups to be vaccinated. For decision makers, the implementation of the influenza vaccination spanning from pandemic to post pandemic was interpreted as a strategic opportunity to protect susceptible persons against the matched viral strain of pandemic influenza virus that was expected to continue to circulate as a seasonal influenza strain. This led some policymakers to justify that susceptible groups be offered vaccines to enjoy the protective effects of the vaccination, at least to some extent. This finding resonates with the agenda of the WHO that recommends the vaccination of people in high risks groups during post pandemic, since possible circulation of the pandemic virus as seasonal influenza cannot reliably be predicted [29]. The limitation to this sort of justification was that in Malawi and Ghana, there was scant data, and with poor surveillance, it was difficult to ascertain whether the pandemic viral strain was in fact circulating as seasonal influenza.

Without adequate information and understanding among policymakers on what might constitute “reasonable coverage” of the influenza vaccine, most policymakers not directly involved in influenza vaccination decisions perceived that, should the virus return, the influenza vaccination strategy implied indirect risk reduction associated with herd immunity. In this study, a number of policymakers made explicit assumptions that 10% coverage would be sufficient to establish herd immunity and so reduce risk. However, key literature on herd immunity supports 75–90% vaccination coverage to achieve herd immunity against influenza [30]. For example, one study reported herd immunity for influenza to be 85%, proven by immunizing school children with a single dose of a monovalent A(H3N2) inactivated vaccine so to reduce the disease burden in the unvaccinated population [13]. In another study in nursing homes, vaccination of more than 70% of residents resulted in herd immunity [31]. This corresponds to a study by researchers in Japan, where more than 80% vaccination coverage against influenza in school children between 1962 and 1987 led to a significant reduction in mortality in the elderly and adults [14].

Our results indicate that the success of vaccine use will depend on a range of factors, including clarity of the pandemic influenza preparedness plans and response in terms of influenza surveillance, risk communication, prevention and containment. All these could inform the significance of influenza vaccine coverage in terms of herd immunity or other perceived benefits, increase acceptance levels, and ensure timely vaccination. This suggests the need for defined goals for vaccination based on different variables. For example, WHO SAGE recommended that health care workers get vaccinated first to protect the integrity of the health system. However, where vaccines are limited, a simulation model suggests that vaccinating 20% of school children would be more likely to reduce influenza related mortality in the elderly than would be the case by vaccinating 90% of persons of age 64 or older [32]. Another study has shown that vaccinating healthy adults yields very modest effects in reducing influenza symptoms and working days lost in the general population, including among pregnant women [33]. Vaccinating the elderly and at risk adults is unlikely to establish indirect protective effects because these groups represent a small percentage of the population among whom the virus spreads. The attack rates for the elderly and at risk adults are relatively low and the vaccine efficacy maybe reduced due to compromised immune systems due to age [34], declining vaccine induced antibody titres, or poor health. Children older than 2 years will respond better to the vaccine than the elderly, who have a declining immunological function due to aging. It is thus important to target children when there are limited doses of vaccine coverage, in order to establish secondary effects in the population, so to ensure that many older people enjoy protection offered by the children’s role in vaccination.

Policymakers expressed concern that members of target groups were unwilling to receive vaccines due to concerns about vaccine safety and religious beliefs. This suggests that there was not enough information about vaccination, during or after the pandemic. This observation corresponds to suggestions that understandings of the heightened risk will lead to people taking action to protect their wellbeing [35]. Through the use of Information, Education and Communication, Ghana was able to tackle vaccination refusal and the sub-optimal uptake was reported to improve to 100%. In contrast, Malawi opted to deploy the police and force high-risk groups to be vaccinated, both on the basis that people were reluctant to volunteer for vaccination and to avoid vaccines been thrown away. Forcing people to take the vaccine supports the view that authorities aspired to satisfy vaccine donors’ requirements for high influenza vaccination coverage. Health providers who received the vaccine were required to use their vaccine supplies [36]. According to Garoon and Duggan ([37], see also Tyler, [38]), the use of force has clear political ramifications. The approach adopted in Malawi raises ethical concerns and tarnished the reputation of public health enforcement officers, while damaging how vaccination policies are viewed [25]. According to Kotalik [39], more information on the benefits and burden of vaccines would set the stage for a more successful voluntary vaccination programme, thus avoiding an ethically problematic mandatory programme. Effective communication is critical for gaining public trust and participating in community measures to contain infection, including vaccination [26].

Despite the important findings, this study had methodological limits related to interviews and data analysis. The inclusion of lay people would have enhanced understanding the multiple factors affecting community vaccine coverage and acceptance. In addition, despite attempts to recruit a representative sample, some crucial policymakers directly involved in writing the pandemic influenza preparedness plans for both Ghana and Malawi were unavailable or failed to consent to participate in the study. This may have had an effect on the generalizability of the findings. There was also a potential for recall bias since the study was conducted a year after the deployment of influenza vaccination programme and 2 years after the pandemic had subsided. The use of computer-assisted qualitative data analysis software (CAQDAS) such as NVivo often creates a distance between the researcher, respondents and the data which can arguably distort data analysis [40]. However, this was resolved through the first author’s familiarity with the empirical data and the second author’s checking of the data, codes and themes created by the software. This data-checking process ensured that the analysis and findings do not suffer from inter-coder unreliability. Reliability was further enhanced by the author’s familiarity with the African context. However, future research that replicates our method in other African countries or repeats our study with a random sample can improve the validity of our findings.

## Conclusion

Vaccines provide good protection against pandemic influenza if administered at the earliest possible time of the outbreak, preferably within 4 to 6 months of outbreak, and if a large population of people are targeted to achieve herd immunity. This study illustrates the difficulty in achieving this when donated vaccines do not arrive on time and in sufficient amounts. The delays experienced by Ghana and Malawi significantly affected the levels of acceptability of vaccines among targeted groups, and informed misunderstandings about vaccine coverage, which most policymakers perceived to be sufficient to establish herd immunity and reduce the risk of further influenza. These barriers can be addressed by developing stronger national influenza preparedness plans to respond to pandemic influenza, which incorporate the rapid procurement of the vaccine supplies and delivery to the points of vaccination, and guidelines for information, education and communication to avoid sub-optimal uptake. Delays in vaccines can also be tackled if governments engage in advance contracts and agreements with pharmaceutical companies for supplies of vaccines as soon as they become available. This will reduce unnecessary reliance on donations. In general, pandemic planning needs to take into account the realities of vaccine availability, timing, coverage, and refusals, and the role of herd immunity, while paying attention to political influences that divert the goals of an effective vaccination programme. Most importantly, to deal with pandemic influenza more effectively, non-pharmaceutical interventions including increased surveillance and the implementation of such policies as quarantine, border control and hygiene practices remain important as the mainstay methods of dealing with a pandemic influenza.
